# The use of healthcare services and disabling chronic pain: results from the cross-sectional population-based Andalusian Health Survey

**DOI:** 10.1093/eurpub/ckae079

**Published:** 2024-05-15

**Authors:** Rocío Cáceres-Matos, Eugenia Gil-García, Soledad Vázquez-Santiago, Andrés Cabrera-León

**Affiliations:** Faculty of Nursing, Physiotherapy and Podiatry, University of Seville, Seville, Spain; Faculty of Nursing, Physiotherapy and Podiatry, University of Seville, Seville, Spain; Faculty of Nursing, Physiotherapy and Podiatry, University of Seville, Seville, Spain; Virgen Macarena University Hospital, Seville, Spain; Andalusian School of Public Health, Cuesta del Observatorio, Granada, Spain; Biomedical Research Consortium in Epidemiology and Public Health Network (CIBERESP), Madrid, Spain; Institute of Biomedical Research ibs.GRANADA, Granada, Spain

## Abstract

**Background:**

Several factors seem to be related to the use of healthcare services, and chronic pain (CP) is among these characteristics. The objective is to describe the number of visits to a doctor’s surgery or emergency rooms, and the periods of hospitalization; to identify characteristics associated with frequent healthcare use, including disabling chronic pain (DCP) and non-disabling chronic pain (n-DCP).

**Methods:**

Representative population-based cross-sectional study of 6569 people older than 16 years from southern Spain was collected. The frequency of visits to a doctor’s surgery or emergency rooms and periods of hospitalization were defined as at or above the 90th percentile. Binary logistic regression analyses were conducted separately on women and men to identify characteristics associated with being frequent visitors.

**Results:**

People with DCP are more frequent visitors to a doctor’s surgery and emergency rooms and endure longer periods of hospitalization compared to people with n-DCP and without pain. In logistic regression models, people with DCP are twice as likely to over-visit a doctor’s surgery; to endure longer periods of hospitalization and more visits to an emergency room service. No relationship was found in n-DCP.

**Conclusions:**

Disability seems to modulate a greater use of health services among the population with CP, doubling it when compared to n-DCP and n-CP, both in women and men. Understanding the role of disability in the use of healthcare services for individuals with CP allows for the identification of needs and strategies to optimize resources.

## Introduction

Chronic pain (CP) affects approximately 20–40% of people throughout diverse populations around the world.^1^ In Spain, CP prevalence is estimated at 17% (95% CI, 16.88–17.19) affecting around 6.7 million people older than 16 years of age.^2^

Treatment involves multiple modalities provided by one or more healthcare professionals or healthcare services. This is also associated with an increase in medical expenditures and a considerable economic burden for individuals and society.^3^ Although CP is the first reason for medical appointments and their inclusion is essential to reflect the burden on society comprehensively,^4^ only a few studies assessed medical costs that affected people with CP pain directly.^5^ Despite efforts to estimate the associated cost, the exact figures are difficult to determine since global data are not available.^6^

Over the last decade, in Canada, CP cost has represented the three largest sources of healthcare cost, exceeding 20 billion dollars.^7^ The cost is estimated to be over 300 billion euros in Europe, which correspond to around 1.5–3% of Gross Domestic Product.^8^^,^^9^

In Spain, where 96.5% of the population is covered by the National Public Health System, there are no global studies that make it possible to accurately calculate the healthcare cost associated with CP.^10^ However, it is estimated that, including direct and indirect costs, it reaches 16 billion euros per year,^11^ which corresponds to 2–2.5% of Gross Domestic Product.^12^ From the point of view of direct costs, CP accounts for 50% of Primary Care consultations and 8% of pharmaceutical spending, placing it as one of the main health problems.^6^

Cost is related to healthcare visits, hospitalization periods and other therapies.^13^ In fact, some studies found that CP increased healthcare service use^14^ and others consider it as one of the main reasons for medical appointments.^15^

Furthermore, several studies coincide in assessing CP when it causes disability, understanding it as dysfunction, limitation in activity and restriction in social participation.^16^ In Spain, a study carried out by Cabrera-León et al. stated that 11.36% (11.23–11.49) of the population over 16 years of age suffer from disabling chronic pain (DCP).^2^ Disability seems to be a predictor that increases healthcare costs and the number of visits to health services. In this regard, another study carried out in the United States by Stockbridge et al., (2015) found that healthcare expenditure per person increased by 4000 dollars when CP caused disability. This figure rises to more than 13 000 dollars when pain interference is severe, concluding that a severe degree of CP is associated with substantially higher healthcare expenditures.^17^

In addition, several factors have been related to healthcare use levels due to CP,^18^ including social status,^2^ age, marital status,^19^ sex; pain intensity,^20^ comorbidities^19^ and occupational situation.^21^

Therefore, DCP represents a major problem for public health and health systems^13^ given that studies indicate that this population presents a greater use of health services.^22^ The objectives of this study were: (i) to describe the number of visits to a doctor’s surgery; periods of hospitalization; and visits to emergency rooms; and (ii) to identify characteristics associated with frequent healthcare use, including DCP and non-disabling chronic pain (n-DCP).

## Methods

### Design and data collection

This cross-sectional study used data obtained from the last Andalusian Health Survey (EAS in Spanish) 2016 edition which was carried out through face-to-face, population-based home interviews. The EAS was designed to evaluate the health status and the use of health services in Andalusia, which is a southern region of Spain and also the fifth most populated region in Europe, with 8.5 million inhabitants. For the design of this study, Sex and Gender Equity in Research (SAGER) guidelines were followed.

The study sample comprised a regionally representative group of 6569 adults (16 years and over). The sample size was calculated for a confidence level of 95%, an expected population percentage of 50% (P = Q), a margin of error for global estimates of ±1.413, and a design effect of 1.35, resulting in a prefixed sample of 6484 individuals. A multistage stratified sample design was adopted using municipalities, census tracts, households and individuals as sampling units. Province, size of municipality and season of the year were considered strata. Municipalities and census tract were selected in proportion to the population size, households with the same probability by systematic sampling, and interviewees by applying stratification for each size of municipality within provinces and quotas for sex-age.

Finally, the interviewer invited one adult per household to participate. The inclusion criteria of this study were people older than 16 years and residing in Andalusia but not institutionalized (e.g. residences, hospitals, prisons, etc.). The survey obtained a response rate of 70.9%. For more information regarding the Andalusian Health Survey, the Andalusian School of Public Health published a report with the details of the sample and methods.^23^

### Variables

#### Frequent number of visits

To assess the frequency with which participants visited healthcare centres, three variables were created (visits to a doctor’s surgery, hospitalization and emergency room visits) from the following questions: (i) how many times have you seen a doctor (public or private) in the last two weeks?; (ii) how many times have you been hospitalized in the last 12 months? and (iii) how many times did you have to use an emergency service in the last 12 months?

Participants answered each question separately, so ‘frequent visits’ were considered separately in every variable. Regarding the term ‘frequent visits’, although there is a lack of consensus in the literature about this concept, the most used definition is considered as the top 10%. Thus, ‘frequent visits’ were denoted as those reporting at or above the 90th percentile for each healthcare professional or setting.^24^ The rest of the participants were categorized as ‘infrequent visits’.

#### Disabling chronic pain and non-disabling chronic pain

The diagnostic criterion to identify the group with DCP and n-DCP was established from the creation of a variable on CP, according to the individuals who declared that a doctor or a nurse had told them that they suffered from one or more of the following types of pain: ‘angina/chest pain’, ‘back pain’, ‘neck pain’, ‘shoulder pain’, ‘waist pain’, ‘cervical/lower back pain’, ‘fibromyalgia’, ‘migraine/headache/chronic cephalgia/frequent headache’ and/or ‘menstrual pain’. This criterion was used in previous studies in a population with similar characteristics.^2^^,^^25^

From the CP variable, two new variables were created composed of disability and CP: DCP and n-DCP. The disability definition encompasses impairments, activity limitations and participation restrictions.^2^^,^^25^ Regarding impairments (problems with bodily functions/structure), interviewers asked people (at home, face-to-face) whether a doctor or a nurse had told them that they suffered from one of the types of pain in the aforementioned list. Activity limitation and participation restriction were constructed from people who declared that they were limited in their daily activity. DCP was formed of people with CP who suffered from a disability and the variable n-DCP of people who declared that they suffered from CP but were not limited in their daily activity.^2^

#### Number of chronic conditions

The variable number of chronic conditions (not including pain diagnoses) was created using the question that assesses whether a doctor or a nurse had told them that they suffered from any of the following chronic conditions: cancer; diabetes; high blood pressure; cholesterol; inflammatory bowel disease; constipation; stomach/duodenum ulcer; pulmonary chronic disease; asthma; cardiac disease; cardiac failure; chronic skin problems; chronic allergies; anaemia; blood circulation problems; varicose veins; haemorrhoids; stroke; depression; anxiety; other mental problems; hearing impairment; cataracts; arthritis or rheumatism; osteoporosis; liver disease; renal disorder; overflow incontinence; infertility and/or prostate disease.

#### Sociodemographic information and lifestyle variables

A selection of independent variables and potential confounders that were included in the analysis was based on a review of prior literature.^26^^,^^27^ The baseline EAS survey demographics included were age (16–44 years; 45–64 years; +65 years), marital status (widowed; divorced, separated; singled; married), alcohol consumption (never consumed; previously consumed; currently consumed), tobacco consumption (never smoked; previously smoked; currently smoked), type of locality (less than 10 000 inhabitants; 10 000–50 000 inhabitants; more than 50 000 inhabitants, capitals), employment status (employed; unemployed; retired/medical leave; housemaker; student), health insurance (public insurance, state mutuals, private insurance, no medical insurance, and public insurance + private insurance), monthly incomes (<€999; €1000–€1999; >€2000) and highest level of education. The latter was categorized into three categories according to the National Classification of Education (CNED in Spanish) (primary education, secondary education and higher education).^28^

### Ethics

The Andalusian Health Survey was supervised and approved by the review board of the General Secretariat of Quality and Public Health for the Health Ministry of the Andalusian Regional Government, and the Andalusian School of Public Health made the data confidential. In addition, to conduct the secondary analysis of the data of this study, consent was requested from the Bioethics Committee for Research at the Virgin Macarena-Virgin del Rocio University Hospitals (Spain), with the code 0057-N-19.

### Statistical analysis

Sociodemographic and general health characteristics were expressed by mean ($\bar{x}$) with confidence interval (CI) in the continuous variables and using frequency and percentage in the categorical variables. The relationship between each sociodemographic and general health characteristic and frequent healthcare visits was first examined using ORs and 95% CIs. Reference categories were selected based on the hypothesized consideration of a higher risk of belonging to the group with a lower frequency of visits.^24^

A backwards manual logistics regression was used to determine the relationship between sociodemographic or health variables and frequent visits to healthcare services (visits to a doctor’s surgery, hospitalization and emergency room visits). The models were initially adjusted using sociodemographic and health services as a control along with the remaining secondary variables. As no pair of sociodemographic or health variables were correlated at *r* ≥ 0.4, all variables were treated as independent variables rather than as confounders.

The statistical criteria for determining the optimal model included comparing the information criteria. They were measures of goodness of fit, including the Akaike Information Criteria, the Bayesian Information Criteria (BIC), and the adjusted BIC (ABIC), with lower values indicating better fitting models. Taking this into account, variables were manually eliminated until the model with the best fit was identified. Significance was considered to be 5% throughout the statistical analysis (descriptive, bivariate and multivariate). A statistical analysis was conducted using IBM SPSS^®^ version 26.0 (IBM Corp., Armonk, NY, USA).

## Results

### Characteristics of the sample

A total of 6569 participants answered the survey (50.8% were women and 49.2% were men). The mean age of women was 46.78 ± 18.15 years and men had an average age of 46.52 ± 18.34 years. In addition, table 1 shows data on age, marital status, highest level of education, monthly incomes, type of locality, employment status, number of chronic conditions and health insurance.

**Table 1:** Sociodemographic and health characteristics of participants

	Women (*n* = 3338)			Men (*n* = 3231)		
Variables	n-CP	n-DCP	DCP	n-CP	n-DCP	DCP
	(*n* = 2687)	(*n* = 187)	(*n* = 464)	(*n* = 2918)	(*n* = 91)	(*n* = 222)
	%(95% CI)	%(95% CI)	%(95% CI)	%(95% CI)	%(95% CI)	%(95% CI)
Age						
16–44 years	53.7(51.8–55.6)^a^	46.5(39.4–53.6)^a^	25.2(21.4–29.2)^a^	52.3(50.5–54.1)^a^	33.0(23.3–42.7)^a^	18.9(13.7–24.1)^a^
45–64 years	29.3(27.6–31.0)	33.2(26.5–39.9)	40.3(35.8–44.8)	30.2(28.5–31.9)	37.4(27.5–47.3)	40.5(34.0–47.0)
+65 years	17.0(15.6–18.4)	20.3(14.5–26.1)	34.5(30.2–38.8)	17.5(16.1–18.9)	29.7(20.3–39.1)	40.5(34.0–47.0)
Marital Status						
Widowed	9.5(8.4–10.6)^a^	12.3(7.6–17.0)^a^	18.1(14.6–21.6)^a^	4.0(3.3–4.7)^a^	8.8(3.0–14.6)^a^	12.2(7.9–16.5)^a^
Divorced, separated	7.3(6.3–8.3)	7.0(3.3–10.7)	8.4(5.9–10.9)	5.1(4.3–5.9)	6.6(1.5–11.7)	5.9(2.8–9.0)
Single	29.5(27.8–31.2)	33.2(26.5–39.9)	14.2(11.0–17.4)	36.0(34.3–37.7)	22.0(13.7–30.7)	14.4(9.8–19.0)
Married	53.7(51.8–55.6)	47.6(40.4–54.8)	59.3(54.8–63.8)	54.9(53.1–56.7)	62.6(52.7–72.5)	67.6 (61.4–73.8)
Highest level education						
Primary studies	56.8(54.9–58.7)^a^	56.1(49.0–63.2)^a^	78.2(74.4–82.0)^a^	57.3(55.5–59.1)^a^	63.7(53.8–73.6)^a^	72.5(66.6–78.4)^a^
Secondary studies	26.2(24.5–27.9)	26.7(20.4–33.0)	13.0(9.9–16.1)	26.7(25.1–28.3)	18.7(10.7–26.7)	17.6(12.6–22.6)
Higher studies	16.9(15.5–18.3)	17.1(11.7–22.5)	8.9(6.3–11.5)	16.0(14.7–17.3)	17.6(9.8–25.5)	9.9(6.0–13.8)
Monthly incomes						
<999 €	41.4(39.5–43.3)^a^	46.3(39.2–53.4)^a^	56.6(52.1–61.1)^a^	38.9(37.1–40.7)^a^	43.8(33.6–54.0)^a^	68.9(62.8–75.0)^a^
1000–1999 €	46.9(45.0–48.8)	39.0(32.0–46.0)	39.2(34.8–43.6)	48.5(46.7–50.3)	45.2(35.0–55.4)	23.9(18.3–29.5)
>2000 €	11.8(10.6–13.0)	14.7(9.6–19.8)	4.2(2.4–6.0)	12.6(11.4–13.8)	11.0(0.6–17.4)	7.2(3.8–10.6)
Type of locality						
Less than 10 000 inhabitants	19.6(18.1–21.1)^a^	20.3(14.5–26.1)^a^	21.8(18.0–25.6)^a^	20.1(18.6–21.6)^b^	20.9(12.5–29.3)^b^	20.7(15.4–26.0)^b^
10 000–50 000 inhabitants	29.6(27.9–31.3)	26.2(19.9–32.5)	28.7(24.6–32.8)	29.5(27.8–31.2)	23.1(14.4–31.8)	30.6(24.5–36.7)
More than 50 000 inhabitants	23.0(21.4–24.6)	15.5(10.3–20.7)	19.0(15.4–22.6)	21.8(20.3–23.3)	20.9(12.5–29.3)	18.9(13.7–24.1)
Capitals	27.8(26.1–29.5)	38.0(31.0–45.0)	30.6(26.4–34.8)	28.6(27.0–30.2)	35.2(25.4–45.0)	29.7(23.7–35.7)
Labour status						
Employed	32.3(30.5–34.1)^a^	35.3(28.5–42.1)^a^	17.2(13.8–20.6)^a^	42.5(40.7–44.3)^a^	42.9(32.7–53.1)^a^	19.8(14.6–25.0)^a^
Unemployed	22.8(21.2–24.4)	24.1(18.0–30.2)	17.7(14.2–21.2)	24.4(22.8–26.0)	17.6(9.8–25.4)	18.5(13.4–23.6)
Retired, medical leave	8.7(7.6–9.8)	11.8(7.2–16.4)	25.4(21.4–29.4)	24.1(22.5–25.7)	38.5(28.5–48.5)	60.8(54.4–67.2)
Housemaker	28.6(26.9–30.3)	21.4(15.5–27.3)	37.5(33.1–41.9)	0.0(0.0–0.0)	0.0(0.0–0.0)	0.0(0.0–0.0)
Student	7.6(4.3–10.9)	7.5(3.7–11.3)	2.2(0.0–6.1)	8.9(7.9–9.9)	1.1(0.0–3.2)	0.9(0.0–2.1)
Chronic conditions (not including pain diagnoses)						
0	59.5(57.6–61.4)^a^	21.6(15.7–27.5)^a^	42.8(38.3–47.3)^a^	63.2(61.5–64.9)^a^	34.1(24.4-43.8)^a^	25.2(19.5–30.9)^a^
1	20.6(19.1–22.1)	16.8(11.4–22.2)	21.9(18.1–25.7)	20.6(19.1–22.1)	33.0(23.3–42.7)	22.5(17.0–28.0)
2	9.7(8.6–10.1)	16.6(11.3–21.9)	11.2(8.3–14.1)	9.4(8.0–10.3)	24.2(15.4–33.0)	16.2(11.4–21.0)
≥3	10.2(9.1–11.3)	45.0(37.9–52.1)	24.1(20.2–28.0)	6.8(5.9–7.7)	8.8(3.0–14.6)	36.0(29.7–42.3)
Health Insurance						
Public insurance	92.4 (91.4–93.4)^a^	90.4 (86.2–94.6)^a^	92.9 (90.6–95.2)^a^	92.1 (91.1–93.1)^a^	85.1 (77.8–92.4)^a^	92.1 (88.6–95.6)^a^
State mutuals	2.2 (1.6–2.8)	3.7 (0.9–6.4)	1.5 (0.4–2.6)	3.6 (2.9–4.3)	4.4 (1.9–8.6)	3.4 (1.0–5.8)
Private insurance	1.2 (0.8–1.6)	1.1 (0.4–2.6)	0.4 (0.2–1.0)	1.0 (0.6–1.4)	1.1 (0.0–3.2)	0.5 (0.0–1.4)
No medical insurance	0.1 (0.6–1.4)	0 (0.0–0.0)	0 (0.0–0.0)	0.2 (0.1–0.3)	0 (0.0–0.0)	0 (0.0–0.0)
Public insurance + private insurance	4.1 (3.4–4.9)	12.8 (8.0–17.6)	5.2 (3.2–7.2)	3.1 (2.5–3.7)	7.7 (2.2–13.2)	4.1 (1.5–6.7)

DCP, disabling chronic pain; n-CP, no chronic pain; n-DCP, non-disabling chronic pain.

a Chi-square test *P* values < 0.05.

b Chi-square test *P* values > 0.05.

### Frequent visits to a doctor’s surgery in the last 15 days

The 90th percentile for frequent visits to a doctor’s surgery was set at more than one visit in the last 15 days. Women (n-DCP: 6.3% vs. DCP: 21.0%) and men (n-DCP: 3.1% vs. DCP: 12.6%) with DCP were more likely to be frequent visitors (≥90th percentile) to a doctor’s surgery in the last 15 days than people with n-DCP. In addition, more women than men suffering from DCP are frequent visitors to these services (women: 21% vs. men: 12.6%) (table 2).

**Table 2:** Distribution of healthcare services use among people with n-DCP and DCP, and without chronic pain, stratified by frequent visitors and gender

Women (*n* = 3338)						
Type of HCP^a^	No frequent visits^b^			Frequent visits^c^		
	n-CP	n-DCP	DCP	n-CP	n-DCP	DCP
	%(95% CI)	%(95% CI)	%(95% CI)	%(95% CI)	%(95% CI)	%(95% CI)
Any appointment	83.8(82.2–85.4)	5.3(1.4–9.2)	10.8(7.0–14.6)	72.8(69.6–76.0)	6.3(3.0–12.3)	21.0(15.5–26.5)
Hospitalized	81.0(80.0–82.0)	5.6(2.2–9.0)	13.4(10.1–16.7)	73.9(67.6–80.2)	6.0(0.0–18.0)	20.1(8.9–31.2)
Emergency services	83.7(82.2–85.2)	4.9(1.2–8.6)	11.4(7.8–15.0)	67.3(62.9–71.7)	8.4(1.1–15.7)	24.3(10.2–21.6)

Men (*n* = 3231)						
Type of HCP^a^	No frequent visits^b^			Frequent visits^c^		
	n-CP	n-DCP	DCP	n-CP	n-DCP	DCP
	%(95% CI)	%(95% CI)	%(95% CI)	%(95% CI)	%(95% CI)	%(95% CI)
Any appointment	92.2(91.1–93.3)	2.7(1.2–6.6)	5.1(1.2–9.0)	84.3(81.5–87.1)	3.1(0.0–10.0)	12.6(6.0–19.2)
Hospitalized	90.6(89.5–91.7)	6.6(3.2–10.0)	2.8(0.0–6.3)	84.4(78.2–90.6)	3.2(0.0–18.6)	12.3(0.0–27.1)
Emergency services	91.7(90.6–92.8)	2.5(1.2–6.2)	5.8(2.2–9.4)	82.8(79.1–86.5)	4.7(0.0–13.3)	12.6(4.3–20.9)

DCP, disabling chronic pain; n-CP, no chronic pain; n-DCP, non-disabling chronic pain.

a HCP, healthcare provider.

b Percent below 90th percentile of visits with/without chronic pain.

c Percent at and above the 90th percentile of visits with/without chronic pain.

**Figure 1:**
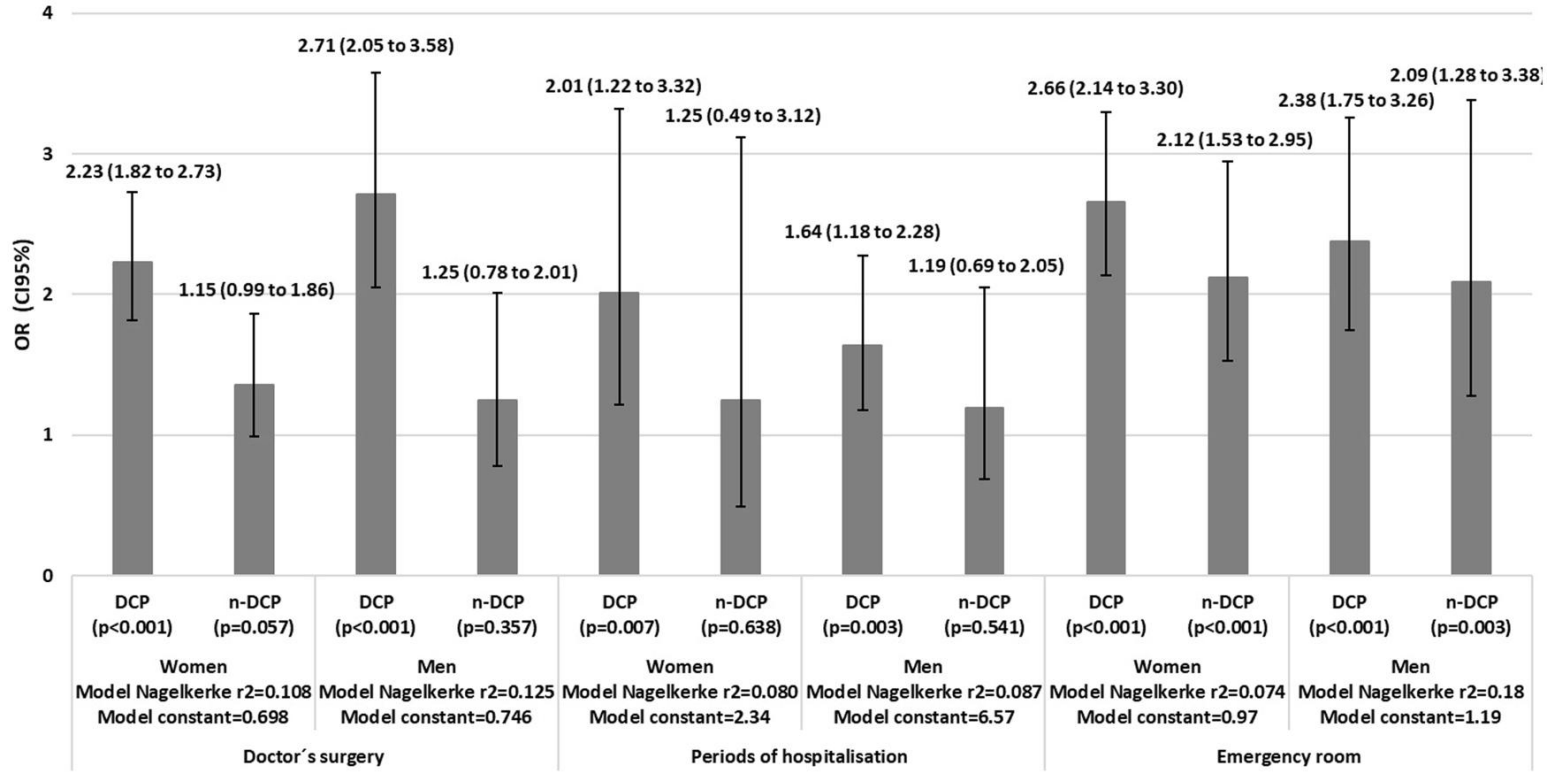
OR of the relationship between Chronic Pain and the use of health services adjusted for age, chronic conditions, marital status, highest level education, monthly incomes, type of locality, labour status, alcohol consumption, tobacco consumption and health insurance

In the logistic regression analysis, DCP was associated with frequent visits to a doctor’s surgery in both women [odd ratio (OR): 2.23, 95% CI: 1.82–2.73]and men (OR: 2.71, 95% CI: 2.05–3.58). On the contrary, n-DCP showed no statistically significant association in both sexes (figure 1).

### Frequent periods of hospitalization in the past year

The 90th percentile for frequent periods of hospitalization in the past year was set at more than one hospitalization period. In both groups, people with DCP showed a higher percentage of frequent hospitalization periods in comparison to people with n-DCP (women: n-DCP: 6.0% vs. DCP: 20.1%; men: n-DCP: 3.2% vs. DCP: 12.3%). In addition, when comparing frequent hospitalization periods between women and men with DCP, women showed a higher frequency of discharges from hospital compared to men (women: 20.1% vs. men: 12.3%; *P* < 0.05) (table 2).

In the logistic regression analysis, DCP was associated with frequent hospitalization periods both in women (OR: 2.01, 95% CI: 1.22–3.32) and men (OR: 1.64, 95% CI: 1.18–2.28). On the contrary, n-DCP showed no statistically significant association in both sexes (figure 1).

### Frequent visits to the emergency room in the past year

The 90th percentile for frequent visits to emergency rooms in the past year was set at more than one visit. In both groups, people with DCP showed a higher percentage of frequent visits to emergency rooms in comparison to people with n-DCP or no pain. In addition, when comparing frequent visits to emergency rooms between women and men with DCP, women showed a higher frequency of discharges from hospital compared to men (women: 24.3% vs. men: 12.6%; *P* < 0.05) (table 2).

In the logistic regression analysis, DCP (women: OR: 2.66, 95% CI: 2.14–3.30 vs. men: OR: 2.38, 95% CI: 1.75–3.26) and n-DCP (women: OR: 2.12, 95% CI: 1.53–2.95 vs. men: OR: 2.09, 95% CI: 1.28–3.38) were associated with frequent visits to emergency rooms (figure 1).

## Discussion

This study is one of the few studies that has analysed the use of health services in the population with CP, despite the fact that pain is considered the first cause of consultation with health services. The results have shown that people with CP more frequently visit a doctor’s surgery, emergency room services and have more hospitalization periods than people without pain, both in women and in men, coinciding with the findings of other similar studies.^29^ In a study carried out by Breivik et al.,^3^ 60% of participants with CP reported that they visited their doctor between two and nine times in the months prior to the start of the study and 10% went at least 10 times.

The results of another study carried out on the Portuguese population found that people with CP visited the doctor twice as often as people without pain.^12^ When comparing CP to other pathologies, another study found that people with pain visited their primary doctor’s surgery up to 5 times more frequently than those people with other diseases.^29^

The results of this study could also be compared with those obtained in another with similar characteristics, carried out in Canada by Mann et al.,^24^ and whose objective was to investigate the role of CP in healthcare visits. However, the aforementioned article only discerned between people who suffer from CP or those who do not suffer from it. This study also includes the role of disability related to CP.

The regression models have shown that disabling CP is associated with a greater use of health services (≥90th percentile), while no differences have been found in those where CP does not cause disability. The results agree with those obtained by Cabrera-León and Cantero-Braojos,^23^ who analysed the previous edition of the Andalusian Health Survey. There seems to be a trend towards a greater use of health services, and it would be disability that would play this modulating role.^31^ The use of general medicine and emergency services was significantly higher in the population with DCP (35.9% and 36.5%), respectively, while hospitalization is almost double (13% in DCP, 7.6% in n-DCP, and 7.1% in n-CP). A greater use of health services was also observed in people with DCP, coinciding with the results observed in the studies developed by Blyth et al.,^22^ and by Häuser et al.^25^ In addition, the use of health services did not differ significantly in the populations with n-DCP and n-CP.^32^

One of the reasons that could explain the greater use of health services among people with DCP is that only a small percentage of these reach the Pain Unit consultations, so they tend to be referred to different medical specialists.^33^ Only 23% of people with CP visit a specialist and 2% are treated by a pain specialist.^34^ This circumstance causes the process that the person must go through before achieving a proper diagnosis and treatment to be long, tortuous, and costly in economic terms. Many patients go through multiple consultations without achieving the desired objective, and as a result the pain they experience starts to affect different areas of their lives and causes disability.^35^

In addition, the analyses of this study have been conducted separately on women and men, unlike that of Mann et al.,^24^ which did not disaggregate by sex. These analyses disaggregated by sex are supported by international regulations and take into account that hyperfrequency is different between women and men. Several researchers have shown that women are more likely to seek healthcare services for pain than men and, for this reason, women could be overrepresented in CP clinics and primary healthcare services.^36^^,^^37^ According to Jonsdottir et al.,^34^ interference of pain in daily life was the strongest predictor among women compared to men. In this sense, healthcare use in relation to CP may not only depend on its severity, but also on interference with daily life. However, results based on sex differences seem to be inconsistent and there are other sociodemographic factors associated with the prediction of a greater use of healthcare.^38^^,^^39^

This study has several strengths, among which it is worth highlighting that it is representative of the population, and its complex design, large sample size, very good response rate and data gathering made it a reliable information source. Measures were taken after providing adequate training to the interviewers to minimize biases related to information, observation, and measures. Finally, with respect to the capacity to generalize the results, although the calibration allows sampling errors and potential selection risks to be reduced, the results of a study carried out by Cabrera-León et al.,^26^ with the same sample demonstrated that there were no differences between the calibrated and non-calibrated estimations. For this reason, we decided not to calibrate.

However, this study also presents a series of associated limitations. The CP variable was created based on self-reporting responses to two questions and may underestimate or overestimate the actual prevalence in a population. In addition, some respondents might not have been diagnosed by a doctor and others, although diagnosed, may not recall the diagnosis. In addition, each person's understanding of the concept of disability is different and subjective. Nevertheless, although self-reported measurements of chronic conditions have shown good levels of correspondence to medical records, this is a limitation to consider. Another limitation is that participants were not asked about lower or upper limbs, which, according to a study carried out by Dueñas et al.,^36^ may account for up to 35.9% of the cases of CP in Spain. However, this study is based on a secondary analysis of data from a population study so this limitation could not be remedied in the analysis design. Finally, since this study follows a cross-sectional design, causality cannot be established.

The results of this study have a significant impact on clinical practice, particularly in economic terms, as it directly addresses the common issue of access to healthcare services for individuals suffering from CP. It is important to note that there is a scarcity of updated studies examining this topic, which underscores the originality and relevance of this work. This study distinguishes itself through its inclusive approach in considering the role of disability associated with pain, thereby broadening our understanding of the complexities in CP management. Furthermore, the evaluation of a population exceeding eight million individuals provides a comprehensive and representative perspective.

These findings lay the groundwork for future research, providing a platform to identify the barriers individuals with CP encounter when accessing healthcare services. This information is crucial for enhancing equity and quality of healthcare for this population. Additionally, the study sheds light on how disability influences the utilization of healthcare services, enabling healthcare professionals to tailor treatments and protocols to the specific needs of each individual. This personalized adaptation is fundamental for maximizing therapeutic outcomes and optimizing resource utilization within the healthcare system.

As future lines of research, we consider it necessary to conduct analyses of these characteristics that allow us to understand the impact that health conditions like CP have on healthcare systems. Another prospective line to consider would be to include factors such as pain intensity or the number of pain sites, which were not taken into account in this study since this data was not available in the Andalusian Health Survey.

## Supplementary Material

ckae079_Supplementary_Data

## Data Availability

Available on demand. Key points People with disabling CP are twice as likely to over-visit a doctor’s surgery, to have more episodes of hospitalization and to make visits to an emergency room service. Disability seems to be the modulating factor that determines a greater use of health services among people with CP. The results of this study have direct implications in clinical practice from an economic perspective, as it addresses a prevalent issue such as the use of healthcare services in individuals suffering from CP.
