# Supplementary material for: The use of healthcare services and disabling chronic pain: results from the cross-sectional population-based Andalusian Health Survey
Source: Eur J Public Health. 2024 May 15;34(4):639–45. doi: 10.1093/eurpub/ckae079 (PMC11293836; doi:10.1093/eurpub/ckae079)
Supplement: ckae079_Supplementary_Data [file ckae079_supplementary_data.docx]

| **Appendix 1. Sociodemographic and general health characteristics stratified by visits to a physician´s office (doctor, specialists, and/or a walk-in-clinic) dichotomised at the 90^th^ percentile of visits in the past 15 days (≤1 and >1)** | | | | | | |
| --- | --- | --- | --- | --- | --- | --- |
|  | **Women (n=3,338)** | | | **Men (n=3,231)** | | |
|  | **No frequent visits***  **% (95% CI)**  **(n=2,332)** | **Frequent visits***  **% (95% CI)**  **(n=1,006)** | **Logistic Regression** | **No frequent visits***  **% (95% CI)**  **(n=2,460)** | **Frequent visits***  **% (95% CI)**  **(n=771)** | **Logistic Regression** |
|  |  |  | **OR (95% CI)** |  |  | **OR (95% CI)** |
| *Age*  16-44 years  45-64 years  +65 years | 53.5 (51.5-55.5)  30.7 (28.8-32.6)  15.8 (14.3-17.3) | 39.9 (36.9-42.9)  31.7 (28.8-34.6)  28.4 (25.6-31.2) | 1.0  2.41 (1.99-2.92)  1.36 (1.17-1.65) | 54.1 (52.1-56.1)  30.3 (28.5-32.1)  15.6 (14.2-17.0) | 34.6 (31.2-38.0)  33.9 (30.6-37.2)  31.5 (28.2-34.8) | 1.0  3.16 (2.56-3.88)  1.75 (1.44-2.12) |
| *Chronic conditions*  0  1  2  ≥3 | 60.5 (58.5-62.5)  19.1 (17.5-20.7)  9.4 (8.2-10.6)  11.0 (9.7-12.3) | 36.6 (33.6-39.6)  22.7 (20.1-25.3)  13.8 (11.7-15.9)  26.9 (24.2-29.6) | 1.0  2.68 (2.18-3.28)  3.37 (2.61-4.35)  5.87 (4.51-7.64) | 66.8 (64.9-68.7)  18.9 (17.4-20.4)  8.5 (7.4-9.6)  5.8 (4.9-6.7) | 37.1 (33.7-40.5)  28.1 (24.9-31.3)  16.0 (13.4-18.6)  18.8 (16.0-21.6) | 1.0  1.97 (1.62-2.39)  2.44 (1.91-3.10)  4.06 (3.31-4.99) |
| *Marital Status*  Married  Widowed  Divorced  Separed  Singled | 52.8 (50.8-54.8)  9.5 (8.3-10.7)  4.3 (3.5-5.1)  3.5 (2.8-4.2)  29.9 (28.8-31.8) | 57.2 (54.1-60.3)  13.9 (11.8-16.0)  4.7 (3.4-6.0)  2.0 (1.1-2.9)  22.3 (19.2-24.9) | 1.0  2.06 (1.47-2.89)  0.78 (0.45-1.34)**  0.77 (0.46-1.29)**  0.63 (0.52-0.75) | 54.4 (52.4-56.4)  3.6 (2.9-4.3)  2.5 (1.9-3.1)  2.8 (2.1-3.5)  36.7 (34.8-38.6) | 8.3 (6.4-10.2)  2.2 (1.2-3.2)  2.5 (1.4-3.6)  25.8 (22.7-28.9)  61.2 (57.8-64.6) | 1.0  1.35 (1.07-1.71)  1.00 (0.69-1.43)**  0.53 (0.32-0.87)  0.69 (0.57-0.82) |
| *Highest level education*  Higher  Primary  Secondary | 17.6 (16.1-19.1)  55.2 (53.2-57.2)  27.2 (25.4-29.0) | 11.7 (9.7-13.7)  70.3 (67.5-73.1)  18.0 (15.6-20.4 | 1.0  1.91 (1.53-2.40)  0.99 (0.77-1.30)** | 16.3 (14.8-17.7)  56.4 (5.4-58.4)  27.3 (25.5-29.1) | 13.3 (10.9-15.7)  65.3 (61.9-68.7)  21.4 (18.5-24.3) | 1.0  1.42 (1.12-1.81)  0.79 (0.96-0.73)** |
| *Monthly incomes*  >2,000 €  <999 €  1,000-1,999 € | 12.0 (10.7-13.3)  42.0 (40.0-44.0)  46.0 (44.0-48.0) | 8.4 (6.7-10.1)  47.7 (44.6-50.8)  43.9 (40.8-47.0) | 1.0  1.62 (1.20-2.19)  1.36 (1.01-1.84) | 12.7 (11.4-14.0)  39.6 (37.7-41.5)  47.7 (45.7-49.7) | 10.5 (8.3-12.7)  46.3 (42.8-49.8)  43.2 (39.7-46.7) | 1.0  1.42 (1.04-1.94)  1.10 (0.81-1.50)** |
| *Type of locality*  Capitals  <10,000 inhabitants  10,000-50,000 inhabitants  >50,000 inhabitants | 30.0 (28.1-31.9)  18.8 (17.2-20.4)  28.8 (27.0-30.6)  22.5 (20.8-24.2) | 25.8 (23.1-28.5)  22.7 (20.1-25.3)  30.4 (27.6-33.2)  21.1 (18.6-23.6) | 1.0  1.40 (1.13-1.73)  1.23 (1.01-1.49)  1.09 (0.88- 1.35)** | 29.7 (27.9-31.5)  19.2 (17.6-20.8)  28.9 (27.1-30.7)  22.2 (20.6-23.8) | 26.1 (23.0-29.2)  23.2 (20.2-26.2)  31.1 (27.8-34.4)  19.6 (16.8-22.4) | 1.0  1.38 (1.09-1.74)  1.23 (0.99-1.52)  1.01 (0.79-1.28)** |
| *Labour status*  Employed  Unemployed  Medical leave/retired  Housemaker  Student | 33.7 (31.8-35.6)  22.6 (20.9-24.3)  8.7 (7.6-9.8)  26.8 (25.0-28.6)  8.2 (7.1-9.3) | 22.7 (20.1-25.3)  21.2 (18.7-23.7)  17.1 (14.8-19.4)  35.4 (32.4-38.4)  3.7 (2.5-4.9) | 1.0  1.39 (1.12-1.73)  2.92 (2.27-3.75)  1.96 (1.61-2.39)  0.67 (0.46-0.98) | 43.7 (41.7-45.7)  25.0 (23.3-26.7)  22.2 (20.6-23.8)  0.0 (0.0-0.0)  9.0 (7.9-10.1) | 32.2 (28.9-35.5)  20.1 (17.3-22.9)  42.4 (38.9-45.9)  0.0 (0.0-0.0)  5.3 (3.7-6.9) | 1.0  1.09 (0.87-1.37)**  2.59 (2.13-3.15)  Excluded  0.80 (0.56-1.15)** |
| *Alcohol Consumption*  Never consumed  Previously consumed  Currently consumer | 27.7 (27.9-29.5)  36.3 (34.3-38.3)  36.0 (34.1-37.9) | 37.6 (34.6-40.6)  36.1 (33.1-39.1)  26.3 (25.6-29.0) | 1.0  0.73 (0.61-0.87)  0.54 (0.45-0.65) | 11.3 (10.0-12.6)  28.7 (26.9-30.5)  60.0 (58.1-61.9) | 15.7 (13.1-18.3)  29.2 (26.0-32.4)  55.1 (51.6-58.6) | 1.0  0.73 (0.56-0.84)  0.66 (0.52-0.84) |
| *Tobacco consumption*  Never smoked  Previously smoked  Currently smoker | 61.0 (59.0-63.0)  10.8 (9.5-12.1)  28.7 (26.9-30.5) | 65.1 (62.2-68.0)  11.2 (9.3-13.1)  23.7 (21.1-26.3) | 1.0  0.97 (0.76-0.94)  0.97 (0.76-1.24)** | 47.2 (45.2-49.2)  17.9 (16.4-19.4)  34.9 (33.0-36.8) | 38.2 (34.8-41.6)  31.3 (28.0-34.6)  30.5 (27.3-33.7) | 1.0  2.16 (1.77-2.65)  1.08 (0.89-1.31)** |
| Health Insurance  Public insurance  State mutuals  Private insurance  No medical insurance  Public and private insurance | 91.7 (90.6-92.8)  2.0 (1.4-2.6)  1.8 (1.3-2.3)  0.2 (0.0-0.38)  4.3 (3.5-5.1) | 92.6 (91.0-94.2)  2.3 (1.4-3.2)  0.8 (0.2-1.4)  0.0 0 (0.0-0.0)  4.2 (3.0-5.4) | 1.0  0.98 (0.91-1.04)**  0.88 (0.74-0.98)**  1.08 (1.11-1.42)**  1.01 (0.95-1.07)**  0.99 (0.89-1.01)** | 90.1 (88.9-91.3)  4.7 (3.9-5-5)  1.2 (0.8-1.6)  0.0 (0.0-0.0)  4.0 (3.2-4.8) | 92.5 (90.6-94.4)  3.3 (2.0-4.6)  0.9 (0.2-1.6)  0.2 (0.0-0.5)  3.1 (2.8-3.4) | 1.0  0.92 (0.86-0.99)**  0.88 (0.72-1.06)**  1.08 (0.95-1.22)**  1.01 (0.94-1.10)**  0.99 (0.92-1.08)** |
|  | Model Nagelkerke r^2^ = 0.108  Model constant = 0.698 | | | Model Nagelkerke r^2^ = 0.125  Model constant = 0.746 | | |
| 1. denotes reference category   *Chi-Square test  **Chi-square test >0.05 | | | | | | |

| **Appendix 2. Sociodemographic and general health characteristics stratified by periods of hospitalisation dichotomised at the 90^th^ percentile of hospitalisation periods in past year (≤1 and >1)** | | | | | | |
| --- | --- | --- | --- | --- | --- | --- |
|  | **Women (n=3,338)** | | | **Men (n=3,231)** | | |
|  | **No frequent visits***  **% (95% CI)**  **(n=3,089)** | **Frequent visits***  **% (95% CI)**  **(n=249)** | **Logistic Regression** | **No frequent visits***  **% (95% CI)**  **(n=3,077)** | **Frequent visits***  **% (95% CI)**  **(n=154)** | **Logistic Regression** |
|  |  |  | **OR (95% CI)** |  |  | **OR (95% CI)** |
| *Age*  18-44 years  45-64 years  +65 years | 48.8 (47.0-50.7)**  31.9 (30.3-33.5)**  19.4 (18.0-20.8)** | 56.6 (50.4-62.8)**  20.5 (15.5-25.5)**  22.9 (17.7-28.1)** | 1.0  0.28 (0.19-0.43)  0.54 (0.36-0.82) | 50.5 (48.7-52.3)  31.0 (29.4-32.6)  18.5 (17.1-19.9) | 29.2 (22.0-36.4)  33.1 (25.7-40.5)  37.7 (30.0-45.4) | 1.0  0.98 (0.71-1.35)**  1.81 (1.30-2.51) |
| *Chronic conditions*  0  1  2  ≥3 | 54.2 (52.4-56.0)  20.2 (18.8-21.6)  10.5 (9.4-11.6)  15.1 (13.8-16.4) | 42.2 (36.1-48.3)  19.3 (14.4-24.2)  13.7 (9.4-18.0)  24.9 (19.5-30.3) | 1.0  2.77 (1.81-4.23)  3.04 (1.82-5.08)  7.02 (4.53-10.9) | 61.2 (59.5-62.9)  20.7 (19.3-22.1)  10.1 (9.0-11.2)  8.0 (7.0-9.0) | 29.9 (22.7-37.1)  27.9 (20.8-35.0)  14.9 (9.3-20.5)  27.3 (20.3-34.3) | 1.0  1.23 (0.86-1.75)**  1.67 (1.18-2.51)  2.13 (1.53-2.96) |
| *Marital Status*  Married  Widowed  Divorced  Separed  Singled | 53.7 (51.9-55.5)**  27.8 (26.2-29.4)**  3.2 (2.6-3.8)**  4.6 (3.9-5.3)**  10.6 (9.5-11.7)** | 58.6 (52.5-64.7)**  24.5 (19.2-29.8)**  1.2 (0.0-2.6)**  2.4 (0.4-4.3)**  13.3 (9.1-17.5)** | 1.0  1.14 (0.77-1.70)**  0.48 (0.21-1.11)**  0.35 (0.11-1.11)**  0.81 (0.59-1.10)** | 55.7 (53.9-57.5)  4.6 (3.9-5.3)  2.5 (1.9-3.1)  2.7 (2.1-3.3)  34.5 (32.8-36.2) | 61.7 (54.0-69.4)  7.8 (3.7-12.0)  1.3 (0.5-3.1)  3.9 (0.8-7.0)  25.3 (18.4-32.2) | 1.0  1.55 (0.83-2.89)**  0.47 (0.11-1.94)**  (1.30 (0.56-3.06)**  0.66 (0.45-0.97) |
| *Level of education*  Higher  Primary  Secondary | 15.6 (14.3-16.9)  59.8 (58.1-61.5)  24.6 (23.1-26.1) | 18.1 (13.3-22.9)  59.4 (53.3-65.5)  22.5 (17.3-27.7) | 1.0  0.86 (0.61-1.22)**  0.79 (0.53-1.19)** | 15.7 (14.4-17.0)**  58.2 (56.5-60.0)**  26.0 (24.5-27.5)** | 13.6 (8.2-19.0)**  63.6 (56.0-71.2)**  22.7 (16.1-29.3)** | 1.0  1.26 (0.78-2.04)**  1.01 (0.58-1.75)** |
| *Monthly incomes*  >2,000 €  <999 €  1,000-1,999 € | 10.7 (9.6-11.8)**  45.6 (43.8-7.4)**  43.7 (42.0-45.4)** | 12.5 (8.4-16.6)**  42.5 (36.4-48.6)**  45.0 (38.8-51.2)** | 1.0  0.88 (0.56-1.41)**  0.80 (0.50-1.27)** | 12.2 (11.0-13.4)**  46.6 (44.8-48.4)**  41.1 (39.4-42.8)** | 11.5 (6.5-16.5)**  46.0 (38.0-53.9)**  42.5 (34.7-50.3)** | 1.0  1.10 (0.59-2.05)**  1.05 (0.56-1.95)** |
| *Type of locality*  Capitals  <10,000 inhabitants  10,000-50,000 inhabitants  >50,000 inhabitants | 28.3 (26.7-29.9)  20.2 (18.8-21.6)  29.3 (27.7-30.9)  22.2 (20.7-23.7) | 34.5 (28.6-40.4)  16.9 (12.2-21.6)  28.5 (22.9-34.1)  20.1 (15.1-25.1) | 1.0  0.68 (0.47-1.00)**  0.80 (0.57-1.10)**  0.74 (0.52-1.06)** | 28.7 (27.1-30.3)  20.1 (18.7-21.5)  29.5 (27.9-31.1)  21.7 (20.2-23.2) | 31.2 (23.9-38.5)  22.7 (16.1-29.3)  27.9 (20.8-35.0)  18.2 (12.1-24.3) | 1.0  1.05 (0.67-1.64)**  0.87 (0.57-1.33)**  0.77 (0.48-1.24)** |
| *Labour status*  Employed  Unemployed  Medical leave/retired  Housemaker  Student | 30.9 (29.3-32.5)  21.7 (20.2-23.2)  10.9 (9.8-12.0)  29.5 (27.9-31.1)  6.9 (6.0-7.8) | 23.7 (18.4-29.0)  28.1 (22.5-33.7)  14.9 (10.5-19.3)  27.7 (22.1-33.3)  5.6 (2.7-8.5) | 1.0  1.69 (1.18-2.42)  1.77 (1.15-2.72)  1.22 (0.85-1.75)**  1.06 (0.58-1.93)** | 41.6 (39.9-43.3)  24.4 (22.9-25.9)  25.8 (24.3-27.3)  0.0 (0.0-0.0)  8.2 (7.2-9.2) | 27.9 (20.8-35.0)  13.0 (7.7-18.3)  51.9 (44.0-59.8)  0.0 (0.0-0.0)  7.1 (3.0-11.2) | 1.0  0.79 (0.46-1.36)**  3.00 (2.05-4.39)  Excluded  1.30 (0.66-2.55)** |
| *Alcohol Consumption*  Never consumed  Previously consumed  Currently consumer | 30.3 (28.7-31.9)**  36.1 (34.4-37.8)**  33.6 (31.9-35.3)** | 35.5 (29.6-41.4)**  37.5 (31.5-43.5)**  27.0 (21.5-32.5)** | 1.0  0.69 (0.50-0.96)  0.89 (0.65-1.20)** | 12.4 (11.2-13.6)  28.4 (26.8-30.0)  59.2 (57.5-61.0) | 12.3 (7.1-17.5)  37.0 (29.4-44.6)  50.6 (42.7-58.5) | 1.0  0.86 (0.51-1.43)**  1.30 (0.77-2.22)** |
| *Tobacco consumption*  Never smoked  Previously smoked  Currently smoker | 45.4 (43.6-47.2)**  34.4 (32.7-36.1)**  20.2 (18.8-21.6)** | 38.3 (32.3-44.3)**  39.0 832.9-45.1)**  22.7 (17.5-27.9)** | 1.0  0.78 (0.51-1.20)**  2.28 (1.58-3.13) | 62.6 (60.9-64.3)**  10.4 (9.3-11.5)**  26.9 (25.3-28.5)** | 57.4 (49.6-65.2)**  17.3 (11.3-23.3)**  25.3 (18.4-32.2)** | 1.0  1.03 (0.75-1.39)**  1.80 (1.26-2.59) |
| *Health Insurance*  Public insurance  State mutuals  Private insurance  No medical insurance  Public and private insurance | 88.4 (87.3-89.5)  2.0 (1.5-2.5)  3.6 (2.9-4.3)  0.0 (0.0-0.0)  6.0 (5.2-6.8) | 92.7 (89.5-95.9)  2.2 (0.4-4.0)  0.9 (0.0-2.1)  0.1 (0.0-0.5)  4.1 (1.6-6.6) | 1.0  1.19 (1.06-1.34)**  0.92 (0.73-1.16)**  0.96 (0.79-1.16)**  1.01 (0.91-1.11)**  0.97 (0.87-1.08)** | 88.3 (87.2-88.3)  5.8 (5.0-6.6)  3.2 (2.6-3.8)  0.0 (0.0-0.0)  2.6 (2.0-3.2) | 92.1 (87.8-96.4)  3.5 (0.6-6.4)  0.8 (0.4-1.2)  0.2 (0.0-0.4)  3.4 (0.5-6.3) | 1.0  0.92 (0.81-1.05)**  1.01 (0.70-1.46)**  0.97 (0.75-1.26)**  1.18 (1.01-1.38)**  0.94 (0.81-1.08)** |
|  | Model Nagelkerke r^2^ = 0.080  Model constant = 2.34 | | | Model Nagelkerke r^2^ = 0.087  Model constant = 6.57 | | |
| 1. denotes reference category   *Chi-Square test  **Chi-square test >0.05 | | | | | | |

| **Appendix 3. Sociodemographic and general health characteristics stratified by emergency room visits dichotomised at the 90^th^ percentile of visits in the past year (≤1 and >1)** | | | | | | | |
| --- | --- | --- | --- | --- | --- | --- | --- |
|  | **Women (n=3,338)** | | | **Men (n=3,231)** | | | |
|  | **No frequent visits***  **% (95% CI)**  **(n=2,683)** | **Frequent visits***  **% (95% CI)**  **(n=655)** | **Logistic Regression** | **No frequent visits***  **% (95% CI)**  **(n=2,738)** | | **Frequent visits***  **% (95% CI)**  **(n=493)** | **Logistic Regression** |
|  |  |  | **OR (95% CI)** |  |  |  | **OR (95% CI)** |
| *Age*  18-44 years  45-64 years  +65 years | 48.5 (46.6-50.4)  32.4 (30.6-34.2)  19.2 (17.7-20.7) | 53.1 (49.3-56.9)  25.3 (22.0-28.6)  21.5 (18.4-24.6) | 1.0  1.40 (1.14-1.72)  0.98 (0.78-1.22)** | 49.0 (47.2-50.9)**  31.7 (30.0-33.4)**  19.3 (17.8-20.8)** | | 51.9 (47.5-56.3)**  28.0 (24.0-32.0)**  20.1 (16.6-23.6)** | 1.0  1.20 (0.96-1.50)**  1.02 (0.79-1.31)** |
| *Chronic conditions*  0  1  2  ≥3 | 55.9 (54.0-57.8)  19.8 (18.3-21.3)  10.4 (9.2-11.6)  13.8 (12.5-15.1) | 42.7 (38.9-46.5)  23.8 (20.5-27.1)  11.9 (9.4-14.5)  21.5 (18.4-24.6) | 1.0  1.55 (1.22-1.98)  1.69 (1.24-2.30)  3.01 (2.25-4.02) | 62.1 (60.3-63.9)  20.5 (19.0-22.0)  9.9 (8.8-11.0)  7.5 (6.5-8.5) | | 46.7 (42.3-51.1)  23.9 (20.1-27.6)  12.6 (9.7-15.5)  16.8 (13.5-20.1) | 1.0  1.42 (1.13-1.78)  1.49 (1.13-1.98)  2.25 (1.80-2.83) |
| *Marital Status*  Married  Widowed  Divorced  Separed  Singled | 54.7 (52.8-56.6)**  10.5 (9.3-11.7)**  4.4 (3.6-5.2)**  3.1 (2.4-3.8)**  27.3 (25.6-29.0)** | 51.8 (48.0-55.6)**  12.3 (9.8-14.8)**  4.6 (3.0-6.2)**  2.6 (1.4-3.8)**  28.8 (25.3-32.3)** | 1.0  1.24 (0.94-1.63)**  1.10 (0.73-1.68)**  0.88 (0.51-1.50)**  1.11 (0.91-1.36)** | 57.3 (55.4-59.2)  4.5 (3.7-5.3)  2.3 (0.5-4.1)  2.6 (2.0-3.2)  33.3 (31.5-35,1) | | 49.1 (44.7-53.5)  5.7 (3.7-7.7)  3.2 (1.6-4.8)  3.7 (2.0-5.4)  38.3 (34.0-42.6) | 1.0  1.46 (0.95-2.25)**  1.64 (0.94-2.89)**  1.64 (0.96-2.80)**  1.34 (1.10-1.65) |
| *Level of education*  Higher  Primary  Secondary | 15.0 (13.6-14.4)  60.1 (58.2-62.0)  24.9 (23.3-26.5) | 19.2 (16.1-22.2)  58.5 (54.7-62.3)  22.3 (19.1-25.5) | 1.0  1.37 (1.03-1.84)  1.23 (0.89-1.71)** | 16.1 (14.7-17.5)**  57.8 (56.0-59.6)**  26.0 (24.2-27.6)** | | 12.7 (9.8-15.6)**  62.2 (57.9-66.5)**  25.1 (21.3-28.9)** | 1.0  0.76 (0.60-0.96)  0.70 (0.53-0.91) |
| *Monthly incomes*  >2,000 €  <999 €  1,000-1,999 € | 11.1 (9.9-12.3)  42.5 (40.6-44.4)  46.4 844.5-8.3) | 10.0 (7.7-12.3)  49.4 (45.6-53.2)  40.7 (36.7-44.5) | 1.0  1.26 (0.91-1.82)**  0.97 (0.69-1.38)** | 12.6 (11.4-13.8)**  40.9 (39.1-42.7)**  46.5 (44.6-48.4)** | | 9.8 (7.2-12.4)**  43.1 (38.7-47.5)**  47.1 (42.7-51.5)** | 1.0  1.36 (0.92-2.01)**  1.30 (0.88-1.92)** |
| *Type of locality*  Capitals  <10,000 inhabitants  10,000-50,000 inhabitants  >50,000 inhabitants | 27.8 (26.1-29.5)  19.9 (18.4-21.4)  29.5 (27.8-31.2)  22.8 (21.2-24.4) | 32.5 (28.9-36.1)  20.2 (17.1-23.3)  28.4 (24.9-31.9)  18.9 (15.9-21.9) | 1.0  0.87 (0.68-1.11)**  0.82 (0.66-1.03)**  0.71 (0.56-0.91) | 28.7 (27.0-30.4)**  20.2 (18.7-21.7)**  29.3 (27.6-31.0)**  21.8 (20.3-23.3)** | | 29.8 (25.8-33.8)**  20.2 (16.7-23.7)**  29.4 (25.4-33.4)**  21.5 (17.9-25.1)** | 1.0  0.95 (0.72-1.25)**  0.99 (0.77-1.27)**  0.89 (0.67-1.17)** |
| *Labour status*  Employed  Unemployed  Medical leave/retired  Housemaker  Student | 30.8 (29.1-32.5)**  21.4 (19.8-23.0)**  11.1 (9.9-12.3)**  29.9 (28.2-31.6)**  6.8 (5.8-7.8)** | 28.4 (24.9-31.9)**  25.3 (22.0-28.6)**  11.9 (9.-14.4)**  27.5 (21.1-30.1)**  6.9 (5.0-8.8)** | 1.0  1.29 (1.02-1.63)  1.17 (0.87-1.57)**  0.99 (0.80-1.25)**  1.09 (0.76-1.57)** | 42.2 (40.4-44.0)  23.7 (22.1-25.3)  26.4 (24.7-28.1)  0.0 (0.0-0.0)  7.6 (6.6-8.6) | | 33.9 (29.7-38.1)  24.7 (20.9-28.5)  30.4 (26.3-34.5)  0.0 (0.0-0.0)  11.0 (8.3-13.8) | 1.0  1.30 (1.01-1.68)  1.43 (1.13-1.82)  ----------  1.79 (1.27-2.51) |
| *Alcohol Consumption*  Never consumed  Previously consumed  Currently consumer | 30.2 (28.5-31.9)**  37.1 (35.3-38.9)**  32.6 (30.8-34.4)** | 32.7 (29.1-36.3)**  32.8 (29.2-36.4)**  34.8 (31.2-38.4)** | 1.0  0.81 (0.66-1.00)**  0.99 (0.80-1.22)** | 12.3 (11.1-13.5)  28.5 (26.8-30.2)  59.1 (57.3-60.9) | | 12.6 (9.7-15.5)  30.4 (26.3-34.5)  57.0 (52.6-61.4) | 1.0  1.04 (0.76-1.44)**  0.94 (0.70-1.27)** |
| *Tobacco consumption*  Never smoked  Previously smoked  Currently smoker | 62.8 (61.0-64.6)**  10.4 (9.2-11.6)**  26.8 (25.1-28.5)** | 59.9 (56.1-63.7)**  13.3 (10.7-15.9)**  26.8 (23.4-30.2)** | 1.0  1.05 (0.86-1.28)**  1.35 (0.03-1.76) | 46.5 (44.6-48.4)  19.9 (18.4-21.4)  33.6 (31.8-35.4) | | 36.6 (32.3-40.9)  27.8 (23.8-31.8)  35.6 (31.4-40.0) | 1.0  1.78 (1.40-2.28)  1.35 (1.10-1.69) |
| *Health Insurance*  Public insurance  State mutuals  Private insurance  No medical insurance  Public and private insurance | 89.3 (88.1-90.5)  2.4 (1.8-3.0)  1.5 (1.0-2.0)  0.0 (0.0-0.0)  6.7 (5.8-7.6) | 93.1 (91.2-95.0)  2.2 (1.1-3.3)  1.0 (0.0-3.4)  0.1 (0.0-3.4)  3.6 (2.2-5.0) | 1.0  1.65 (0.51-2.66)**  1.16 (1.08-3.26)**  0.99 (0.93-1.07)**  1.05 (0.98-1.13)**  1.03 (0.86-1.23)** |  | 90.0 (88.9-91.1)  4.1 (3.2-4.7)  1.0 (0.9-1.1)  0.4 (0.2-0.6)  4.3 (1.8-6.8) | 92.3 (90.0-94.7)  3.5 (1.9-5.1)  1.0 (0.1-1.9)  0.1 (0.0-0.3)  3.1 (1.6-4.6) | 1.0  1.03 (0.84-1.26)**  1.16 (0.98-1.34)**  0.99 (0.99-1.17)**  0.60 (0.53-0.68)**  0.44 (1.10-1.22)** |
|  | Model Nagelkerke r^2^ = 0.074  Model constant = 0.97 | | | Model Nagelkerke r^2^ = 0.18  Model constant = 1.19 | | | |
| 1. denotes reference category   *Chi-Square test  **Chi-square test >0.05 | | | | | | | |
